# Viral load monitoring for people living with HIV in the era of test and treat: progress made and challenges ahead – a systematic review

**DOI:** 10.1186/s12889-022-13504-2

**Published:** 2022-06-16

**Authors:** Minh D. Pham, Huy V. Nguyen, David Anderson, Suzanne Crowe, Stanley Luchters

**Affiliations:** 1 Burnet Institute, Melbourne, Australia; 2 Department of Epidemiology and Preventive Medicine, Faculty of Medicine Nursing and Health Science, Monash University, Melbourne, Australia; 3 Health Innovation and Transformation Centre, Federation University, Victoria, Australia; 4 School of Medicine and Dentistry, Griffith University, Brisbane, Australia; 5 Department of Microbiology, Faculty of Medicine Nursing and Health Science, Monash University, Melbourne, Australia; 6 Central Clinical School, Faculty of Medicine Nursing and Health Science, Monash University, Melbourne, Australia; 7 Centre for Sexual Health and HIV & AIDS Research, Harare, Zimbabwe; 8 Department of Public health and Primary care, Ghent University, Ghent, Belgium

**Keywords:** HIV, Viral load monitoring, Decentralisation, Low and middle-income countries

## Abstract

**Background:**

In 2016, we conducted a systematic review to assess the feasibility of treatment monitoring for people living with HIV (PLHIV) receiving antiretroviral therapy (ART) in low and middle-income countries (LMICs), in line with the 90-90-90 treatment target. By 2020, global estimates suggest the 90-90-90 target, particularly the last 90, remains unattainable in many LMICs. This study aims to review the progress and identify needs for public health interventions to improve viral load monitoring and viral suppression for PLHIV in LMICs.

**Methods:**

A literature search was conducted using an update of the initial search strategy developed for the 2016 review. Electronic databases (Medline and PubMed) were searched to identify relevant literature published in English between Dec 2015 and August 2021. The primary outcome was initial viral load (VL) monitoring (the proportion of PLHIV on ART and eligible for VL monitoring who received a VL test). Secondary outcomes included follow-up VL monitoring (the proportion of PLHIV who received a follow-up VL after an initial elevated VL test), confirmation of treatment failure (the proportion of PLHIV who had two consecutive elevated VL results) and switching treatment regimen rates (the proportion of PLHIV who switched treatment regimen after confirmation of treatment failure).

**Results:**

The search strategy identified 1984 non-duplicate records, of which 34 studies were included in the review. Marked variations in initial VL monitoring coverage were reported across study settings/countries (range: 12–93% median: 74% IQR: 46–82%) and study populations (adults (range: 25–96%, median: 67% IQR: 50–84%), children, adolescents/young people (range: 2–94%, median: 72% IQR: 47–85%), and pregnant women (range: 32–82%, median: 57% IQR: 43–71%)). Community-based models reported higher VL monitoring (median: 85%, IQR: 82-88%) compared to decentralised care at primary health facility (median: 64%, IRQ: 48-82%). Suboptimal uptake of follow-up VL monitoring and low regimen switching rates were observed.

**Conclusions:**

Substantial gaps in VL coverage across study settings and study populations were evident, with limited data availability outside of sub-Saharan Africa. Further research is needed to fill the data gaps. Development and implementation of innovative, community-based interventions are required to improve VL monitoring and address the “failure cascade” in PLHIV on ART who fail to achieve viral suppression.

## Background

In 2014, UNAIDS launched the 90-90-90 treatment target: by 2020, 90% of all people living with HIV (PLHIV) know their status, 90% of all people diagnosed with HIV infections receive antiretroviral therapy (ART) and 90% of all people on ART have suppressed viral load [1]. Evidence from a large body of research demonstrates that ART can improve the health of PLHIV and stop onward transmission [2–4]. Modelling data suggests achieving the 90-90-90 target, which means having 73% of all PLHIV virally suppressed, coupled with scaling up other prevention measures, could reduce new HIV infection and HIV-related death worldwide by 90% between 2010 and 2030 [5]. On the basis of this evidence, universal HIV testing and treatment in a broader health system approach has been identified as a key strategy for ending the epidemic by 2030, even in the absence of an effective vaccine [6].

In low and middle-income countries (LMICs), there has been an intense focus on scale-up of ART programs to increase treatment coverage in PLHIV through decentralisation of HIV services [7–9]. Global estimates indicate a significant increase in the number of PLHIV with access to ART over the past decade: from 6.4 (95% uncertainty intervals (UIs) 5.9–6.4) million in 2009 to 25.4 (95% UIs 24.5–25.6) million by the end of 2019, with 95% of people taking ART residing in LMICs [10, 11]. Decentralised HIV care, with services delivered at primary care level, is feasible and can improve patient access and adherence to HIV treatment whilst maintaining high quality of care in various settings [12–14]. However, there is legitimate concern about the sustainability of ART programs in LMICs without substantial financial and technical support from international donors and implementing partners [15, 16]. An increase in ART coverage means a greater need for treatment monitoring to ensure program effectiveness and efficiency. Treatment monitoring, however, requires substantial infrastructure, supply chain management system, financial and human resources, which are already scarce in many decentralised settings [17].

In 2016, we conducted a systematic review to assess the feasibility of ART monitoring for PLHIV in the context of decentralised HIV treatment and care in LMICs [18]. The conclusions were as follows: (1) There were limited published data on the coverage of treatment monitoring, particularly viral load (VL) monitoring for PLHIV on ART in real-world settings; (2) There were potential gaps in the coverage and quality of treatment monitoring services across countries and regions; (3) There was an urgent need to strengthen treatment monitoring (particularly VL monitoring) to improve the HIV continuum of care in LMICs; and (4) Point-of-care (POC) diagnostics could play an important role in scaling up and improving the quality of treatment monitoring for PLHIV on ART in decentralised settings.

Despite continuous gains in access to testing and treatment since 2015, the ambitious 90-90-90 treatment target, particularly the last 90, remains unattainable in many LMICs [19]. Reaching the new 95-95-95 target in 2030 will be an uphill task for these countries. To make meaningful progress towards this treatment goal will require much greater use of VL monitoring – the gold standard approach for assessment of HIV treatment at individual, program and population levels. The objectives of this study were to: (1) outline the current state of research and progress on VL monitoring for PLHIV on ART in decentralised settings in LMICs, and (2) identify gaps in research and the needs for public health interventions to improve VL monitoring coverage and quality of HIV treatment programs for PLHIV in LMICs.

## Methods

The study was designed and reported in accordance with the Preferred Reporting Item for Systematic Reviews and Meta-Analyses (PRISMA) statement [20]. The initial search strategy developed for the 2016 review was used to search MEDLINE for articles published from 1 to 2015 (end date of prior search) to 31 May 2020. The search strategy is available online [18]. In addition, a PubMed search was conducted using following search terms: “HIV”, “routine viral load”, and “viral load monitoring” for the same period (1 December 2015–31 May 2020) with an updated search conducted in August 2021. Bibliographies of all articles included for full-text review were searched manually to identify relevant studies.

Prior to July 2021, WHO recommended that patients with elevated VL (≥ 1000 copies/ml) should be given enhanced adherence consultation (EAC) followed by a repeat VL test within 3–6 months of the initial VL. If the repeat VL remains greater than 1000 copies/ml, treatment failure is confirmed and patients should be switched to second-line ART [21]. For the purpose of this review, the following outcomes of interest were pre-defined: *Primary outcome*: Initial VL monitoring (the proportion of PLHIV active on first-line ART (the main treatment option available at decentralised settings in LMICs) and eligible for VL monitoring who receive a VL test for treatment monitoring purposes). *Secondary outcomes* include: (i) Follow-up VL monitoring (the proportion of PLHIV on ART who received a follow-up VL test following an initial elevated VL as defined by included studies); (ii) Confirmation of treatment failure (the proportion of PLHIV on ART with two consecutive elevated VL results among those who received a follow-up VL test) and; (iii) Switching treatment regimen (the proportion of PLHIV on ART switching treatment regimen among those with confirmed treatment failure as defined in included studies). 

To be included in this review, studies must meet the following inclusion criteria: (1) was conducted in LMICs; (2) involved PLHIV receiving first-line ART and eligible for VL monitoring; (3) involved decentralised HIV treatment and care, defined as having treatment and treatment follow-up provided in non-hospital settings – primary health care facilities or community-based health care services; and (4) reported the primary outcome of interest. Only studies published in English were included.

Data were extracted electronically using a pre-defined data extraction form. The following information was extracted: study details (first author and year of publication, study design and data source, study population, study sites/locations, study period and study objective); VL testing model; external funding and/or technical supports received and outcome of interests. Information on treatment regimen and VL threshold for definition of elevated VL and treatment failure were also extracted, if reported. Data on outcomes of interest are presented by time point or follow-up period as per the included studies. Outcomes by model of care (community-based vs. primary health care) and subgroups of study population (e.g., children, adolescent, pregnant women, etc.) are extracted (if reported) and presented to enable data comparison. Descriptive statistics were applied to describe the outcomes of interest across countries and study settings, study populations and models of care.

## Results

**Fig. 1:**
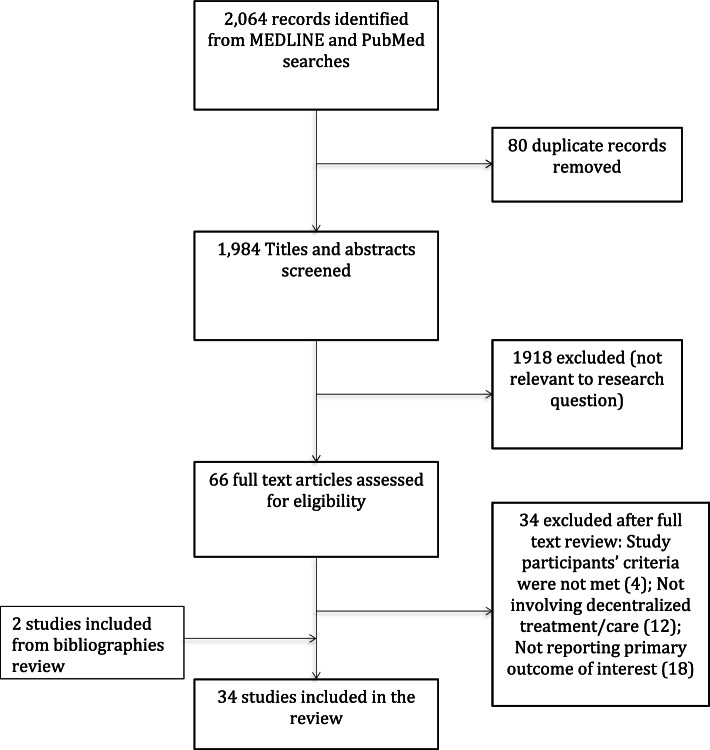
Study selection process

The search strategy yielded 1984 records after removal of duplicates. Screening titles and abstracts identified 66 studies for full-text examination, of which 32 studies met the inclusion criteria and were included. Bibliography review identified two additional studies, making a total of 34 studies included in the review (Fig. 1).

Twenty-nine of the 34 included studies were conducted in and/or had data collected from sub-Saharan Africa (SSA) countries, including South Africa [22–32], Zimbabwe [33–36], Rwanda [37, 38], Lesotho [39], Swaziland [40], Senegal [41], Malawi [42], Democratic Republic of Congo [43], Kenya [44], Mozambique [45] and Uganda [46–48]. Twenty studies were designed to assess VL monitoring or VL cascade in PLHIV on ART with long-term follow-up of > = 12 months and large sample sizes (> 500). Most (28/34) studies used routinely collected program data in real-world settings, with seven studies focusing on children, adolescent and young people aged 0–24 years, four studies on pregnant women. One study reported data from 18 LMICs in Asia and Africa over 12 year-period (2006–2018) [49] and one study was conducted among HIV infected incarcerated people [50] at three correctional complexes in South Africa and Zambia (Table 1).

**Table 1:** Characteristics of included studies

First author, year	Study design/data sources	Study participant (N enrolled)	Study sites/ Location	Study period	Study objective	VL testing model	External Funding/ tech. partner	Outcomes
Apollo, T.; 2021 [36]	Retrospective study using routinely collected program data from the national electronic patient monitoring system (ePMS) of Zimbabwe	PLHIV enrolled and remained in ART care between August 2004 – Jan 2017, for at least 12 months. (N = 392,832)	529 high volume (525 public & 4 private) health facilities providing HIV treatment and care services across Zimbabwe	2004–2018	To determine the number (%) of PLHIV on ART progressed along the HIV cascade	A combination of centralised VL testing with near POC VL (at 25 s-level health facilities)	None mentioned	- Initial VL monitoring - Follow-up VL monitoring - Confirmation of treatment failure - Switching treatment regimen
Brazier, E.; 2021 [49]	Observational study using data from the international epidemiology Databases to evaluate AIDS (IeDEA) research consortium	ART-naïve patients enrolled in HIV care at study sites between 2006 and 2018 who had at least 9 months potential follow-up after ART initiation (N = 292,380)	Selected IeDEA HIV care and treatment sites in 18 low and lower middle-income countries which contribute patient data prior to and after national adoption of Treat all policy	2006–2018	To evaluate the effect of Treat All policy adoption on pre-ART CD4 testing and VL monitoring after ART initiation	Not reported	None mentioned	- Initial VL monitoring
Haghighat, R.; 2021 [28]	Longitudinal cohort study using data collected from routine (paper-based/electronic) clinical records and interviews with study participants	Adolescents aged 10–19 years who had initiated ART at study sites between 03/2014-09/2015 (N = 1080)	52 healthcare facilities within a health district of the Eastern Cape, South Africa (decentralised public healthcare system)	2014–2017	To examine the progression of adolescents on ART along the HIV cascade of care (from treatment initiation to viral suppression)	Not reported	None mentioned	- Initial VL monitoring
Woldesenbet, S. A.; 2021 [29]	Cross-sectional study using data extracted from medical records (conducted as part of national survey to monitor prevention of MTCT of HIV program)	HIV-positive pregnant women aged 15–49 years who initiated ART before pregnancy or during pregnancy and received ART for > = 3 months (N = 8112)	Public, primary healthcare facility of 52 districts from 9 provinces in South Africa (SA)	Oct – Nov 2019	To evaluate the coverage of maternal viral load monitoring in South Africa	Laboratory-based VL testing (Roche & Abbott assays) at 17 public laboratories in SA	None mentioned	- Initial VL monitoring
Herce, M.E.; 2020 [50]	Prospective cohort study using data collected by the study team	HIV positive incarcerated people aged > = 18 years, and expected to remain incarcerated for > = 30 days at study sites (N = 975)	10 correctional units at three correctional complexes in South Africa (Johannesburg & Breede River) and Zambia (Lusaka)	2017–2018	To report clinical outcome of a prospective cohort of incarcerated people with HIV who were offered universal test and treat intervention and follow-up	Laboratory-based VL testing at central laboratory facilitated/paid for by the study	Department of International Development UK (DFID) & Swedish International Development Agency (Sida)	- Initial VL monitoring
Mshweshwe-Pakela, N.; 2020 [30]	Retrospective study using data extracted from patient charts and national electronic laboratory service database	People > = 18 years who had a positive HIV test recorded at study sites between 1st Jan-31 July 2017 (N = 826)	10 public sector health facilities in Ekurhuleni District, South Africa	2017–2018	To examine factors associated with retention in care & VL suppression among HIV patients at study sites	Not reported	None mentioned	- Initial VL monitoring
Nakalega, R.; 2020 [47]	Cross-sectional study using data extracted from paper and electronic ART registries and patient treatment card	PLHIV who were receiving (and on ART for at least 6 months) at study sites between Jan-Dec 2017 (N = 414)	Eight (08) primary health care centres providing HIV treatment and care services in Gomba district, rural Uganda	Oct-Nov 2018	To describe factors associated with non-uptake of VL testing among PLHIV in Gomba, Uganda	Laboratory-based VL testing (COBAS Amplipera/AmpliTaq) at Central public health laboratory	None mentioned	- Initial VL monitoring
Opito, R.; 2020 [48]	Retrospective cohort study using routinely collected data from HIV service registries and electronic medical record system	All patients who were newly diagnosed HIV positive at study site between Jun 2017 and May 2018 (N = 580)	An HIV (TASO) clinic in Tororo district, Eastern Uganda	2017– 2018	To explore the outcome of the implementation of HIV test and treat policy at study site	Laboratory-based VL testing at by central public health laboratories through a hub system	None mentioned	- Initial VL monitoring
Ya, S.S.T.; 2020 [53]	Retrospective study using routinely collected integrated HIV care (IHC) program data extracted from the central electronic database of the national AIDS program	All adults and paediatric PLHIV who started 1st line ART between Jan 2016 and Dec 2017 (N = 91,290	49 IHC clinics in 37 townships of 05 regions in Myanmar	2016–2018	To describe programmatic performance and outcomes of routine VL testing in PLHIV newly initiating 1st line ART in IHC program in Myanmar	Laboratory-based VL testing at the central public health laboratory in Mandalay, Myanmar	The international Union Against Tuberculosis and Lung Disease (the Union)	- Initial VL monitoring - Follow-up VL monitoring - Confirmation of treatment failure - Switching treatment regimen
Moudachiro, R.; 2020 [43]	Retrospective cohort study using routine program data collected from electronic database and patient records	Patients aged > 15 years, stable on ART, transferred to community-based centres (CBC) for 3-monthly ART supply during the study period (N = 337)	Three decentralised community ART refill centres in Kinshasa, Democratic Republic of Congo (DRC)	Jan 2015 – Jun 2017	To assess VL coverage and sustained, viral suppression and retention in care among ART patients at CBC at 6, 12, 18 months after transfer	Yearly clinical and VL monitoring at nearest health facility	CBC set up by Médecins Sans Frontières (MSF)operated by Ministry of Health of DCR	- Initial VL monitoring
Iwuji, C.C.; 2020 [22]	Cohort study conducted as part of a demographic surveillance and population-based HIV survey, using routine program data captured in an electronic patient register (Tier.Net)	Patients aged > 15 years who initiated ART at study sites during the study period (N = 29,384)	17 public sector clinics in rural Hlabisa sub-district of KwaZulu-Natal, South Africa (SA)	Jan 2010 – Dec 2016	To evaluate the implementation of VL monitoring guidelines in rural KwaZulu-Natal, SA	Laboratory-based VL testing done at district hospital with plasma samples collected at & transported from clinics daily	None mentioned	- Initial VL monitoring - Follow-up VL monitoring - Confirmation of virological failure - Switching treatment regimen
Nguyen, A.T.; 2020 [55]	Prospective cohort study using patient-level data collected directly by the study team	Adult patients who initiated ART at HIV clinics in remote areas during the study period (N = 578)	43 clinical sites in six northern provinces of Vietnam	Jun 2017 – Apr 2018	To assess the feasibility of DBS use for VL monitoring in remote areas of Vietnam where routine VL monitoring was not available	DBS samples collected at study sites and sent to central laboratory for testing	Global Fund to fight AIDS, Tuberculosis and Malaria	- Initial VL monitoring - Follow-up VL monitoring - Confirmation of virological failure
Kehoe, K.; 2020 [23]	Retrospective cohort study using routinely collected program data contributed to an international epidemiology database (IeDEA-SA)	Patients aged 16–85 years who initiated ART from Jan 2004 onwards & enrolled into adherence club in Khayelitsha, Cape Town, SA between Jan 2011 and Dec 2016 (N = 8058)	Adherence club (AC) operated at community venues in Khayelitsha, Cape Town, SA	Jan 2011 – Dec 2017	To describe VL completeness and assess proportion of elevated VL and confirmed virologic failure among patients entering AC in Khayelitsha, Cape Town, SA	VL testing done in batches for each AC	None mentioned (AC model was first piloted by MSF in 2007 and adopted by the local health authority in 2011)	- Initial VL monitoring - Follow-up VL monitoring - Confirmation of virological failure
Thinn, K.K.; 2019 [52]	Retrospective cohort study using data extracted from patient-specific treatment card at clinics and laboratory information management system at National Health Laboratory (NHL)	All patients initiated on ART at study sites between Jan–Dec 2017 under the National AIDS Program (NAP) (N = 567)	6 ART clinics operated at peripheral public health facilities under NAP in Yangon region, Myanmar	2017–19	To assess the uptake of VL monitoring after ART initiation and factors influencing the implementation of routine VL monitoring	Laboratory-based VL testing (Abbott m200rt) done at NHL using fresh plasma samples (patients referred or blood samples sent to NHL)	The International Union Against TB and Lung Disease (the Union); London School of Hygiene and Tropical Medicine, UK	- Initial VL monitoring - Follow-up VL monitoring - Confirmation of virological failure
Tapera, T.; 2019 [33]	Retrospective cohort study using routinely collected data of a community adolescent treatment supporter (CATS) program to improve the health and well-being of PLHIV aged 0–24 years	HIV-positive contacts and sexual partners aged < 25 years of index PLHIV identified by CATS between Oct 2017 & Sept 2018 in study sites (N = 1193)	24 selected districts of Zimbabwe implementing the CATS program	2017–19	To assess the effects of CATS program on HIV care cascade outcomes of HIV-positive contacts/sexual partners of index PLHIV	Not reported	Multi-donor/implementing partner: PEPFAR, USAID, CDC, UK Department for International Development (DFID), the Union etc.	- Initial VL monitoring
Nyakura, J.; 2019 [34]	Retrospective cohort study using routine program data collected from health facility registers	Pregnant women living with HIV who have their 1st antenatal care visit at study sites in 2017 & on ART for > = 3 months (N = 1112)	Public health facilities (3 hospitals & 25 rural health clinics in Mazowe, a rural district of Zimbabwe	2017–18	To assess the uptake and turn around time of VL testing among women on option B+	Laboratory-based VL testing done at National Microbiology Reference Laboratory using DBS or venous blood samples sent from health facilities twice a week	None mentioned	- Initial VL monitoring - Follow-up VL monitoring
Nyagadza, B.; 2019 [35]	Review of VL monitoring program using data from routine reports and patients’ record at MSF-supported health facilities	Patients on ART at selected study sites who were eligible for VL monitoring ( > = 6 months on ART) (N = 9456)	10 primary health facilities (HFs) in 5 districts of Manicaland province, Zimbabwe	2016–17	To report MSF experience in supporting VL monitoring scale-up in Zimbabwe	VL testing done at provincial hospital laboratory on whole blood or DBS samples sent from HFs	MSF	- Initial VL monitoring - Follow-up VL monitoring - Confirmation of virological failure - Switching treatment regimen
Nicholas, S.; 2019 [42]	Retrospective cohort using program data (patient-level data captured at study sites)	All patients (all ages) on ART for > 3 months & eligible for VL testing at study sites (N = 21,004*) 396 patients on 2nd ART excluded from this review	Four decentralized clinics (DCs) & one district hospital (DHOS) in the rural Chiradzulu district, southern Malawi	Aug 2013 – Jun 2017	To describe the outcomes of VL monitoring during the first four years of a routine POC VL testing program in real-world settings	On-site point-of-care VL testing (SAMBA I technology; Diagnostics for Real World, UK)	MSF; funding from UNITAID/ MSF	- Initial VL monitoring - Follow-up VL monitoring - Confirmation of virological failure - Switching treatment regimen
Ndagijimana Ntwali, J. D.; 2019 [37]	Retrospective cohort study using routinely collected HIV program data	All patients (all ages) who were initiated on ART at study sites (N = 775)	One public district hospital and one health centre located in the district of Musanze in the Northern province of Rwanda	Jul 2012 – Dec 2016	To examine the use of routine VL testing in patients on ART and describe the management of patients with detectable VL	Laboratory-based VL testing (COBAS Amplipera/AmpliTaq). Whole blood samples were sent to the laboratory for processing & testing	None mentioned	- Initial VL monitoring - Follow-up VL monitoring - Confirmation of virological failure - Switching treatment regimen
Le Roux, K. W.; 2019 [24]	Retrospective study using data obtained through iDART – a hospital pharmacy database	All patients who were initiated on ART at study sites (N = 882)	One district hospital and 11 health clinics in the deeply rural Zithulele, Eastern Cape of South Africa (SA)	Jul 2013 – Jun 2014	To describe the outcomes of innovative interventions to improve the quality of ART programs in rural SA	Not Reported	None mentioned	- Initial VL monitoring
Euvrad, J.; 2019 [25]	Retrospective cohort study using three data sources: primary healthcare information system, provincial health data centre and physical patient folder at health facilities	All patients on ART and in care at primary health facilities in Khayelitsha with an expected VL test monitoring during the study period (N = 21,991)	All primary health facilities in Khayelitsha, Cape Town, SA	Jul 2015 – Jun 2016	To describe the VL cascade from expected (VL test) to reported (VL result) and to estimate success/failure at each step	Laboratory-based VL testing using COBAS Ampliprep/COBAS TaqMan system	Free public ART services including VL testing was provided with support from MSF	- Initial VL monitoring
Cisse, A. M.; 2019 [41]	Cross-sectional study using biological and clinical data collected at study sites	HIV-infected children and adolescents aged 0–19 years receiving follow-up care at decentralised health facilities (N = 601)	All 72 clinics outside of Dakar region providing care for HIV-infected children in Senegal	Mar – Jun 2015	To detect virological failure and test a nationwide sample delivery circuit using DBS	Laboratory-based VL testing using Nucli-SENSEasy Q/Abbott RealTime HIV-1 m2000rt system	None mentioned	- Initial VL monitoring
Namale, G; 2019 [46]	Cross-sectional study using data routinely collected at a research clinic via clinical records and self-reported questionnaire	HIV-positive female sex worker (FSW) aged ≥ 18 years on ART for at least 6 months (N = 584)	A research clinic (Kampala, Uganda) established in 2008 for research on HIV/STIs among FSWs	Jan 2015-Dec 2016	To assess factors associated with virological failure among HIV-positive FSWs in Kampala, Uganda	VL testing performed on plasma using Abbott RealTime HIV-1 PCR assay	PEPFAR, UK Medical Research Council, DFID	- Initial VL monitoring
Sunpath, H.; 2018 [31]	Cross-sectional comparative study (pre and post-intervention) using routine program data collected from national electronic medical record register (TIER.net) and a district health information system	PLHIV actively enrolled for care; had been receiving ART since 2006 for varying period of time and were due for VL testing (N = 9184)	Three (03) publicly operated HIV clinics in rural areas of eThekwini district of Kwa-Zulu Natal, SA	Nov 2016 – Nov 2017	To assess the impact of an intervention program to enhance VL monitoring (VL champion – trained nurse to follow-up on VL testing and clinical management)	Laboratory based VL testing using fresh plasma sample (NHLS)	None mentioned	- Initial VL monitoring
Moyo, F.; 2018 [32]	Retrospective cohort study using data extracted from facility-based medical records and national HIV electronic register	HIV infected mother – infant pairs in three districts of KwaZulu-Natal province, SA (N = 367)	Three districts (eThekwini, uMgungundlovu and uMkhanyakude) with highest burden of HIV in SA	May – Sept 2016	To describe the gaps in prevention of mother to child transmission of HIV at study sites	Not reported	None mentioned	- Initial VL monitoring
Janurag, P. P.; 2018 [54]	Prospective cohort study using patient-level data collected directly by the research team	HIV-infected individuals aged ≥ 16 years from key populations^a^ attending services at study sites (N = 831)	25 local clinics providing HIV services to key populations in Bali, Bandung, Jakarta & Yogyakarta, Indonesia	Sep 2015 – Sep 2016	To report the first year’s results of an intervention project to improve the cascade of HIV care among key populations	Not reported. *(Free VL testing provided at ART initiation and every 6 months thereafter by the project)*	Research project with support from the Australian Department of Foreign Affairs and Trade, WHO and the Indonesian Government	- Initial VL monitoring
Kadima, J.; 2018 [44]	Case-control study conducted using routine program data extracted from patients charts and an electronic database of VL results	Children aged 0–15 years on ART at study sites who received a VL test between Jun 2014 and May 2015 (N = 1272)	5 primary health care facilities (3 sub-country hospital and 2 health centres) at Homabay, Migori and Kisumu counties, Kenya	2014–16	To describe the adoption of routine VL monitoring among patients on ART aged < 15 years in western Kenya	VL samples were collected on-site and sent for central processing/testing at regional laboratory	PEPFAR/CDC, University of California at San Francisco; US	- Initial VL monitoring - Follow-up VL monitoring - Confirmation of virological failure - Switching treatment regimen
Etoori, D.; 2018 [40]	Prospective cohort study using data extracted from patients’ files and electronic VL database	HIV + pregnant women aged ≥ 16 years enrolled into PMTCT option B + at study sites between Jan 2013-June 2014 (N = 665)	One secondary and 8 primary care facilities in Nhlangano health zone, Southern Swaziland	Jan 2013 – Sep 2015	To describe outcomes including VL testing uptake of a feasibility study of PMTCT option B + in the public health sector	VL testing performed using the Biocentric (Bandol, France) multi-manufacturer open platform on plasma samples	PMTCTB + implementation supported by MSF; VL testing funded by UNITAID	- Initial VL monitoring
Copelyn, J.; 2018 [26]	Retrospective cohort study using data obtained from hospital database and patient folders & an electronic database at provincial health data centre	All children aged < 15 who were initiated on ART at a hospital and subsequently down-referred to two primary health clinics (PHCs) during study period (N = 116)	One hospital and two PHCs (within the immediate catchment area of the hospital and < 10 km apart) in Cape Town, SA	Jan 2006 – Dec 2012	To assess the success (clinical and ART outcomes) of a down-referral process in a cohort of young children	Not reported	None mentioned	- Initial VL monitoring
Amzel, A.; 2018 [39]	Retrospective study using program-level data extracted from PEPFAR monitoring, evaluation and reporting	All patients aged 5–24 years who were on ART for 12 months in the three study districts by end of Sep 2017 (N = 11,337)	All health facilities in three districts (Maseru, Mafeteng, Mohale’s Hoek) of Lesotho that offer community-supported interventions for children and adolescents living with HIV on ART	Oct 2016 – Sep 2017	To assess retention, VL coverage & viral suppression among children and adolescents living with HIV on ART in three study districts	Not reported	Multi-donor/ Implementing partners: PEPFAR, Elizabeth Glazer Pediatric AIDS Foundation, Baylor International Pediatric AIDS Initiative	- Initial VL monitoring
Tsondai, P.R.; 2017 [27]	Retrospective cohort study using data from AC registers, patients’ clinic folders and provincial health data centre	Patients on ART enrolled into AC model at study sites between Jan 2011 and Dec 2014 (N = 3216)	100 ACs operated by 15 health care facilities within Cape Town health district, Cape Town, SA	2011–15	To describe outcomes (loss to follow-up, viral rebound) of AC model	Not reported	Bill and Melinda Gates Foundation	- Initial VL monitoring
Swannet, S.; 2017 [45]	Retrospective cohort study using routine program data	All patients (all ages) who had spent > 6 months on 1st line ART (eligible for routine VL) with ≥ one consultation during the study period (N = 43,579)	Six MSF-supported HC in Matupo, Mozambique	2014–15	To describe the VL cascade & examine factors influencing VL testing uptake and results	VL testing done at a hospital laboratory (bioMerieux, NucliSENS EasyQ HIV-1 V2.0). HCs send DBS samples to laboratory for testing	MSF	- Initial VL monitoring - Follow-up VL monitoring - Confirmation of virological failure - Switching treatment regimen
Kyaw, N.T.; 2017 [51]	Retrospective cohort study using routine program data extracted from an electronic database	Non-pregnant patients aged > = 10 years who were initiated on and receive 1st line ART for > = 6 months at study sites (N = 23,248)	33 clinics (ART centres & decentralised sites) under Integrated HIV Care (IHC) program in Mandalay, Sagaing, Magway, Shan & Yangon, Myanmar	Feb 2005 – Jul 2015	To describe the rates of treatment failure and switching to second-line ART in adolescent and adult patients receiving first-line ART under IHC program	Laboratory-based VL testing done at a private laboratory (2009–12) or the public health laboratory in Mandalay (since 2013)	The Union; DFID,	- Initial VL monitoring - Switching treatment regimen
Cyamatare Rwabukwisi, F.; 2016 [38]	Retrospective cohort study using routinely collected data of a community-based accompaniment program (CBAP)	All patients aged < 15 years who were initiated on ART in CBA program at study sites during the study period (N = 277)	All 25 ART facilities offering CBAP in two rural districts of Rwanda	Jan 2005 – Dec 2008	To describe 5-year outcomes of children with HIV enrolled in the CBA program	Not reported	None mentioned	- Initial VL monitoring - Follow-up VL monitoring

^a^ Key populations include: people who inject drug, female sex worker, men who have sex with men and transgender women

**Fig. 2:**
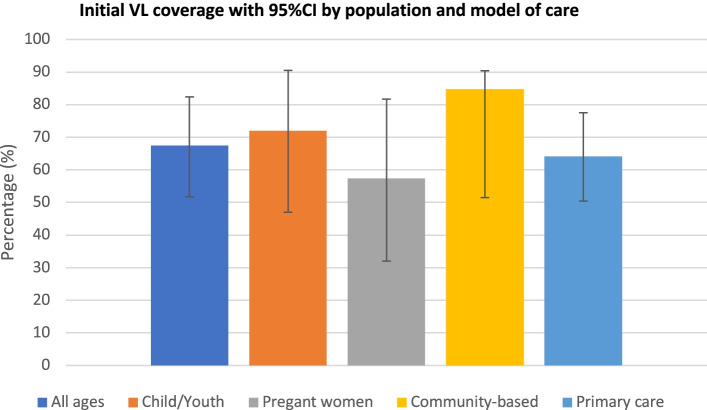
Proportion of patients with a viral load monitoring test within 6-12 months after ART initiation (median and 95% confidence interval)

Wide ranges of VL monitoring coverage in program settings were observed. Studies of adults/patients of all ages, children, adolescents (aged 0–19 years) and young people (aged < 25) years), and pregnant women reported VL coverage of 25–96% (median: 67% interquartile range IQR: 50-84%), 2–94% (median: 72%, IQR: 47-85%) and 32–82% (median: 57%, IQR: 43-71%), respectively. Studies include community-based care models reported higher initial VL monitoring coverage (median: 85%, IQR: 82-88%) compared to decentralised care at primary health facility (median: 64%, IRQ: 48-82%) (Fig. 2). Reported proportions of patients on first-line ART who received a first VL monitoring test within 6–12 months of ART initiation ranged from 12% [49] to 94% [31] (median: 74%, IQR: 46-82%). Similarly, reported annual uptake of VL monitoring varied across countries, from 25% in Zimbabwe [36] to 94% in Kenya [44] (median: 66%, IQR: 47-87%) (Table 2).

**Table 2:** Reported uptake of viral load monitoring, confirmation of treatment failure and switching treatment regimen rates from included studies

First author, year	Follow-up/ Observation period					Notes
		***Initial VL monitoring***^**a**^ ***(IVL)***	***Follow-up VL monitoring***^**b**^ ***(FUVL)***	***Confirmation of virological failure*** ^**c**^	***Switching to 2nd line ART*** ^**d**^	
Apollo, T.; 2021 [36]	2004–2018	99,902/392,832 (25.4%)	6,689/17,970 (37.2%)	4,086/6,689 (61.1%)	1,749/4,086 (42.8%)	- IVL (%) of patients on ART for > = 12 months who had at least one VL test done - Details of EAC (after initial elevated VL ≥ 1000 copies/ml) not provided
Brazier, E.; 2021 [49]	6 months	34,570/292,380 (11.8%)	-	-	-	- Adults (> 19 years): 31,147/260,735 (11.9%) Adolescents (10–19 years): 1,295/11,619 (11.1%) Children (< 10 years): 2128/20,026 (10.6%) - Coverage before and after Treat All policy: Adults (9.1% & 34.7%); Adolescents (9.1% & 26.5%); Children (9.9% & 22.6%)
Haghighat, R.; 2021 [28]	12 months	449/951 (47.2%)	-	-	-	− 951/1,080 have clinical record available; 878/951 (92.3%) had at least one VL result recorded. Among those with any VL data, 51.1% (449/878) and 75.4% (662/878) had their most recent VL recorded in past 12 months and 24 months, respectively.
	24 months	662/951 (69.6%)	-	-	-	
Woldesenbet, S. A.; 2021 [29]	2019	6,542/8,112 (81.7%)	-	-	-	- VL testing recommended for all HIV (+) pregnant women at delivery, on ART at 1st ANC visit or 3 months after ART initiation
Herce, M.E.; 2020 [50]	6 months	269/346 (78%)	-	-	-	- IVL: % individuals with completed VL monitoring scheduled at 6- and 12-months follow-up visits
	12 months	96/149 (64%)	-	-	-	
Mshweshwe-Pakela, N.; 2020 [30]	6 months	455/710 (64.1%)	-	-	-	VL test done between 4 and 8 months after ART initiation
Nakalega, R.; 2020 [47]	12 months	279/414 (67.4%)	-	-	-	- VL testing done at 6 months after ART initiation and 12 months thereafter at national reference Lab
Opito, R.; 2020 [48]	12 months	221/422 (52.4%)	-	-	-	VL monitoring assessed by the presence of VL slip captured in medical record within 12 months of enrolment on test and treat
Ya, S.S.T.; 2020 [53]	6 months	952/1,892 (50.3%)	88/106 (83%)	23/88 (26%)	9/23 (39.1%)	- PLHIV started ART between: (a) 1/1/2016 and 30/6/2017 were scheduled for 12 VL testing; (b) 1/7/2017 and 31/12/2017 were scheduled for 6 months VL testing due to change in policy - Details of EAC (after initial elevated VL ≥ 1000 copies/ml) not provided
	12 months	3,476/6,816 (51%)	273/346 (78.9%)	115/273 (42.1%)	98/115 (85%)	
Moudachirou, R.; 2020 [43]	12 months	118/306 (38.5%)	-	-	-	Yearly VL test done at closest health facility 9–18 months after transfer to PODIs (decentralized community ART centres)
Iwuji, C.C.;2020 [22]	6 months	9,861/24,199 (40.7%)	-	-	-	- IVL time windows: 5–9 months for 6 months and +/- 3 months for 12 & 24 months after ART initiation - FUVL done within 6 months of the initial elevated VL - VL monitoring coverage varied across sites. Optimal monitoring to 12, 24 months were 12%, 6%, respectively - Details of EAC (after initial elevated VL ≥ 1000 copies/ml) not provided
	12 months	7,765/22,807 (34.0%)	-	-	-	
	24 months	4,334/16,965 (25.5%)	658/2135 (30.8%)	408/658 (62%)	141/408 (34.5%)	
Nguyen, T.A.; 2020 [55]	6 months	397/537 (73.9%)	15/59 (25.4%)	11/15 (74.4%)	-	- DBS samples collected during clinic visit 4–10 months after ART initiation - Details of EAC (after initial elevated VL ≥ 1000 copies/ml) not provided
Kehoe, K.; 2020 [23]	4 months	5,340/6,547 (82%)	-	-	-	- Routine VL required at 4 months after entering adherence club (AC) and annually thereafter - Details of EAC (after initial elevated VL ≥ 1000 copies/ml) not provided
	16 months	3,171/3,856 (82%)	-	-	-	
	28 months	1,841/2,170 (85%)	-	-	-	
	40 months	884/1,061 (83%)	-	-	-	
	2011–17	7,136/8,058 (89%)	291/441 (66%)	150/291 (52%)		- Proportion of patients with at least one VL test after AC entry - FUVL done within 12 months after initial elevated VL
Thinn, K.K.; 2019 [52]	24 months	288/498 (57.8%)	8/25 (32%)	6/8 (75%)	-	- Proportions of first routine VL test done within 6–9 months, 9–15 months and after 15 months of ART initiation were: 56/498 (11.2%); 113/498 (22.7%) and 119/498 (23.9%); respectively - Details of EAC (after initial elevated VL ≥ 1000 copies/ml) not provided
Tapera, T.; 2019 [33]	12 months	1,044/1,153 (91%)	-	-	-	- VL test conducted 6–12 months after ART initiation
Nyakura, J.; 2019 [34]	6 months	354/1,112 (32%) - Hospitals: 146/327 (44.6%) - Clinics: 208/785 (26.5%)	13/20 (65%)	-	-	- VL data collected at health facilities up to 6 months post delivery - Proportions of women with VL test done before, at or after delivery, and “unknown time” were: 113/1112 (10%); 124/1,112 (11%) and 117/1,112 (10.5%) - Details of EAC (after initial elevated VL ≥ 1000 copies/ml) not provided
Nyagadza, B.; 2019 [35]	12 months	5,966/9,456 (63%)	281/622 (45.2%)	233/281 (83%)	108/233 (46.4%)	- IVL: patients eligible for VL monitoring who had at least one VL test done during the observation period - FUVL: 23 (8%) had a follow-up VL within 3 months, another 153 (56%) within 3–6 months of initial high VL − 31% of patients with IVL ≥ 1000 copies/ml have at least 1 EAC session documented
Nicholas, S.; 2019 [42]	48 months	17,832/21,004 (85%) - Hospitals: 5,112/6,237 (82%) - Clinics: 13,060/15,163 (86%)	1,277/1,544 (83%) - Hospitals: 79%; - Clinics: 84%;	901/1,277 (70.6%)	434/540 (80%) - Hospitals: 67%; - Clinics: 86%;	- IVL: First VL test at 6 months after ART initiation - Patients eligible for 2nd line ART after having 2 consecutive FUVLs (3 months apart) of ≥ 1000 copies/ml - Details of EAC (after initial elevated VL ≥ 1000 copies/ml) not provided
Ndagijimana Ntwali, J. D.; 2019 [37]	2013	39/152 (25.6%)	-	-	-	- IVL: 12-month VL uptake among patients active on ART - FUVL: coverage among patients active on ART 6 months after their high initial VL - Details of EAC (after initial elevated VL ≥ 1000 copies/ml) not provided
	2014	195/312 (62.5%)	-	-	-	
	2015	347/494 (70.2%)	-	-	-	
	2016	510/547 (93.2%)	-	-	-	
	2013–16	698/775 (90%)	103/117 (88%);	41/103 (40%)	26/41 (63.4%)	
Le Roux, K. W.; 2019 [24]	6 months	480/579 (83%)	-	-	-	- Outcomes of an intervention program to encourage on-time VL testing with reduced clinic visits - VL done within 2–7 months & within 12 months of ART initiation
	12 months	536/579 (92.6%)	-	-	-	
Euvrad, J.; 2019 [25]	60 months	18,450/21,991 (84%)	-	-	-	Routine VL done within window periods: 3–9 months for 1st VL; 9–18 months for second VL; and +/- 6 months for every annual VL expected thereafter
Cisse, A. M.; 2019 [41]	2015	2% (n/N not reported)	-	-	-	- Patients on ART who have at least one documented VL during follow-up - Median time on ART: 21 months (IQR: 1-129)
Namale, G; 2019 [46]	2015–16	477/584 (81.7%)				- VL testing done 6 months after ART initiation and 12 months thereafter
Sunpath, H.; 2018 [31]	6 months	4,889/5,196 (94%)				VL testing rate at 6 and 12 months of an intervention program to improve VL monitoring (baseline rate: 63% (547/864)
	12 months	10,640/11,096 (96%)				
Moyo, F.; 2018 [32]	2016	110/233 (47.2%)	-	-	-	- IVL: % of women (HIV infected mother) received ART who had a documented VL result
Janurag, P. P.; 2018 [54]	12 months	325/457 (71%)	-	-	-	First VL test done around 6 months after ART initiation
Kadima, J.; 2018 [44]	12 months	1,190/1,272 (93.6%)	66/98 (67.3%)	51/66 (77.3%)	9/51 (17.6%)	- Initial routine VL defined as most recent VL received Jun 2014 – May 2015 − 98 cases randomly selected from 442 patients with elevated VL (VL ≥ 1000 copies/ml) - No child received a FUVL within 3 months, only 9/66 (14%) had a FUVL within 6 months of initial elevated VL - Details of EAC (after initial elevated VL ≥ 1000 copies/ml) not provided
Etoori, D.; 2018 [40]	24 months	251/337 (67.3%)	-	-	-	- First VL test done 6–12 months after ART initiation
Copelyn, J.; 2018 [26]	6 months	60/75 (80%)	-	-	-	Documented VLs at 6 & 12 months after down-referral to decentralized care
	12 months	54/75 (72%)	-	-	-	
Amzel, A.; 2018 [39]	12 months	5,365/11,337 (47%)	-	-	-	- Proxy VL coverage indicator: Numerator: Number of patients with a documented VL. Denominator: Number of patients on ART at study sites as of 30/9/2017
Tsondai, P.R.; 2017 [27]	4 months	2,782/3,216 (86.5%)	-	-	-	- VL testing required at 4 months after AC enrolment and every 12 months thereafter
	16 months	1,563/1,846 (84.7%)	-	-	-	
	28 months	490/615 (79.7%)	-	-	-	
Swannet, S.; 2017 [45]	24 months	17,236/34,514 (50%)	1,095/2,600 (42%)	678/1,095 (62%)	212/678 (31.3%)	- IVL: First VL test done during the observation period - VL cut-off: 3000 copies/ml - Only 50% (339/678) patients with 2 consecutive VL ≥ 3000 copies/ml were proposed and approved for 2nd -line ART - Details of EAC (after initial elevated VL ≥ 1000 copies/ml) not provided
Kyaw, N.T.; 2017 [51]	2005–15	7,888/23,248 (34%)	-	-	762/1,032 (74%)	- Target VL monitoring was implemented - IVL: proportion of patient who tested for VL among PLHIV on ART ≥ 6 months
Cyamatare Rwabukwisi, F.; 2016 [38]	60 months	235/277 (84.8%)	25/34 (73%)	-	-	- IVL: a documented VL result within 5 years of ART initiation - FUVL: an additional VL after a VL of ≥ 1000 copies/ml within 5 years of ART initiation - Details of EAC (after initial elevated VL ≥ 1000 copies/ml) not provided

^a^ Initial VL monitoring:

- Proportion of patients on ART who receive VL monitoring test

o Numerator: Number of patients active on 1st line ART who have a VL test for treatment monitoring

o Denominator: Total number of patients retained in care, active on 1st line ART and eligible for VL monitoring

^b^ Follow-up VL monitoring:

- Proportion of patients on ART with an initial unsuppressed VL who receives a follow-up VL test

o Numerator: Number of patients active on 1st line ART who had a follow-up VL test after an initial unsuppressed VL

o Denominator: Total number of patients active on 1st line ART with a documented unsuppressed VL (defined as ≥ 1000 copies/ml unless otherwise noted) and eligible for follow-up VL monitoring test (e.g., have had EAC after initial unsuppressed VL)

^c^ Confirmation of virological failure:

- Proportion of confirmed treatment failure among patients who were suspects of failing treatment

o Numerator: Number of patients on 1st line ART with two documented, consecutive unsuppressed VLs

o Denominator: Number of patients active on 1st line ART who had a follow-up VL test after an initial unsuppressed VL (defined as ≥ 1000 copies/ml unless otherwise noted)

^d^ Switching treatment regimen:

- Proportion of patients switched to 2nd ART after confirmation of virological failure

o Numerator: Number of patients who switched to 2nd line ART after confirmation of virological failure

o Denominator: Total number of patients on 1st line ART with confirmation of virological failure (having two documented, consecutive VL results of ≥ 1000 copies/ml unless otherwise noted)

Of 11 studies from South Africa, four were conducted in Cape Town, reporting relatively high VL coverage of 72–89% [23, 25–27]. Studies with interventions to improve quality of ART program had a VL coverage of > 90% whilst other studies reported a VL range of 25-81%. Of the 17 studies conducted in other SSA countries, 10 studies (59%) reported funding and/or technical support from international donors/implementing partners for implementation of ART and/or VL monitoring programs. Two of four studies from Zimbabwe with donors’ support reported VL coverage at 63% [35] and 91% [33], whilst the others (with no external funding/technical support) reported VL coverage of 32% [34] and 25% [36]. Two studies reported coverage by sites, enabling a comparison between VL monitoring in decentralised care versus centralized/hospital care. A study conducted in Malawi reported slightly higher VL coverage at decentralised clinics (with direct support from Médecins Sans Frontières – MSF) than district hospitals (86% vs. 82%) [42]. In contrast, a study at public health facilities in Zimbabwe reported a significantly lower uptake of VL testing at rural health clinics (26.5%) than hospital settings (44.6%) [34].

Of five studies in Asia, three were conducted in Myanmar using national HIV program data and reported a range of VL coverage from 34% [53] to 57% [52, 55]. Two other studies were conducted in research contexts where VL testing was historically not available: one reported the outcomes of an intervention project to improve the cascade of care among key populations in Indonesia [54], and the other was a trial designed to assess the feasibility of dried blood spot (DBS) use for VL monitoring in remote regions in Vietnam [51]. Free VL testing was provided to study participants, and coverage was 71–73% at 6 months after ART initiation.

Thirteen studies provided data on the proportion of patients on ART receiving a follow-up VL test after an initial elevated VL (follow-up VL monitoring). Overall, the coverage for follow-up VL monitoring was lower than that of initial VL monitoring with nine out of 13 studies reporting gaps of 2–48% points (Table 2). The reported coverage also varies across study settings and countries, from 25 to 88% (median 65.5%, IQR: 38–77%). Eleven studies reported the outcomes of follow-up VL monitoring, and the proportions of patients with two consecutives elevated VL measurements (confirmation of treatment failure) varied from 26 to 83%, with a median of 62% (IQR: 49.5–74.5%). The reported proportion of patients switching to second-line ART after confirmation of treatment failure also varied, ranging from 18 to 85% (median 45%, IQR: 36–71%).

## Discussion

The findings of this review demonstrate an increasing interest in research on VL monitoring and VL cascades for PLHIV receiving treatment at decentralised settings in LMICs over the past six years. Nearly 60% (20/34) of the included studies were specifically designed to evaluate the implementation and/or reported the outcomes of VL monitoring program for PLHIV on ART. Most of these studies were published in 2019–2021. This represents a substantial increase in the number of studies focused on VL monitoring compared to our previous review [18], with only two of 21 included studies assessing the coverage of treatment/VL monitoring services for PLHIV on ART. Recent global estimates showed that whilst the number of PLHIV who know their HIV status and the number of PLHIV who are on treatment increased steadily between 2015 and 2018, the proportion of PLHIV with viral suppression has remained stable with substantial variations across regions and many LMICs lagging behind[19]. Insufficient access to VL testing, lack of appropriate action on VL results and lack of access to second and third ART regimens were key barriers in translating significant gains in knowledge of HIV status and treatment coverage to viral suppression among PLHIV in low-resource settings [56].

Consistent with findings from previous studies, the results of this review show that there are still significant gaps in VL monitoring across countries and regions. Studies that reported high VL monitoring coverage and use of VL results for patients’ management were conducted in settings with one or more of the following: (1) support from international donors and implementing partners; (2) innovative interventions and models of service delivery for decentralisation of HIV treatment and care, such as “hub and spoke”, “differentiated care” or “adherence club”; and (3) research clinics with free HIV services, including VL testing, for participants. From programmatic perspectives, whilst studies conducted in research or program contexts with external funding and technical supports may have limited generalizability, the finding that community-based models of care, implemented by local government or health authorities, can deliver high VL coverage is encouraging, and supports the continued scale-up of decentralisation of HIV treatment and care in LMICs.

LMICs face immense financial and implementation challenges in provision of HIV treatment and routine VL monitoring for PLHIV on ART. External financial and technical supports with a multi-sector approach are crucial for the set-up and delivery of such services, particularly in decentralised settings where VL monitoring was historically unavailable [57]. However, as HIV treatment (and VL monitoring) programs mature, such interventions and supports must be integrated within the local healthcare system to ensure sustainability and long-term gains. Evidence from this review shows that even within a program, clinical sites with more direct support outperformed others in term of service delivery (VL monitoring coverage) and patients’ outcomes (VL suppression) [42]. Therefore, it is critical to strengthen local health system capacity to ensure efficient use of available resources and maintain desirable outcomes when direct external support declines. Operational research is needed to identify cost-effective interventions and best practices to improve VL outcomes for PLHIV in LMICs.

There was also evidence of a narrow geographical focus for research on HIV treatment and VL monitoring programs with most of the data come from SSA countries. Even within SSA, almost all studies included in this review were conducted in Eastern and Southern Africa – the region with the second-highest point estimate of the proportion of people who are virally suppressed among all PLHIV (58%, 95%UIs: 50–66%), behind only high-income countries with well-developed health care systems in Western and Central Europe and North America (64%, 95%UIs: 54–74%) [19]. This raises a concern about the dearth of data from countries and regions with lower level of VL coverage and viral suppression rate. Our study findings call for a renewed focus of financial resources and international research efforts in settings with limited data available and likely lack of progress in improving VL monitoring and viral suppression among PLHIV on ART.

Our findings suggest that research and targeted interventions are needed to improve VL monitoring coverage in vulnerable populations, including children, adolescents and young people, pregnant women and key populations (KPs) living with HIV.

As HIV treatment programs expand in many LMICs, children and adolescents are a priority population for the scale-up of VL monitoring to improve quality of care and treatment outcomes [58]. The startling variation (2–94%) in VL testing coverage among children and adolescents identified herein suggests that in some low-resource settings in LMICs, the health care system is unprepared and unable to address the needs of this vulnerable population. This review found only four studies that report VL monitoring in pregnant women receiving care at decentralised care settings. The findings are in line with those of previous studies [59, 60], suggesting suboptimal VL monitoring in pregnant women living with HIV on ART, with higher coverage in hospital settings [34] and/or programs funded by international donor/implementing partners [40]. Further research is needed to determine an optimal VL monitoring schedule in this population, develop and implement targeted interventions supporting those who receive PMTCT and HIV services at decentralised settings in LMICs. Ad hoc VL testing (e.g., using POC tests) should also be available for targeted VL monitoring as medically indicated or in case turnaround time for laboratory test is too long for meaningful clinical interventions.

Our review highlights the lack of data on VL monitoring for KPs in real-world settings. Only two studies [46, 54] reported the proportion of PLHIV on ART receiving VL monitoring among members of KPs with high coverage. These data, however, have poor generalizability, because levels of dedicated staff and resources would not be sustainable outside the research context. Many LMICs have concentrated HIV epidemics among KPs. Monitoring VL and level of viral suppression in PLHIV who are members of KPs and engage in high-risk behaviours is crucial to determine the trajectory of the epidemic. If those who do not achieve VL suppression and engage in high-risk behaviours are the driving force of HIV transmission in the community [61], then the ultimate goal of reducing population-level HIV incidence and ending the HIV epidemic will not be achieved [62].

Routine VL monitoring for PLHIV on ART is only meaningful if accompanied by the use of VL results for appropriate and timely clinical action. The findings of this review indicate suboptimal uptake of follow-up VL among patients with initial elevated VL (median 66%, IQR: 38–77%); high proportion of confirmed treatment failure among those patients who had a follow-up VL (median 62%, IQR: 50–75%) and a low switching rate among those with confirmed treatment failure (median 45%, IQR: 36–71%). These findings are of particular concern, because they could indicate inadequate EAC and/or a high level of treatment failure due to drug resistance that requires switching the ART regimen. It is known that prolonged treatment regimen failure significantly increases the risk of multiple drug-resistant mutations and compromises the efficacy of second-line ART in HIV-infected children [63], adults [64], and pregnant women [65]. PLHIV on ART who fail to achieve viral suppression enter a “failure cascade” that can jeopardise individual health and the overall effectiveness of HIV programs [66]. Operations research and intervention strategies are needed to address treatment adherence and the failure cascade to preserve the efficacy of first/second-line ART, thereby avoiding exhausting treatment options in LMICs.

This review contributes to a growing body of literature highlighting the importance of VL monitoring and VL cascade analysis for planning, implementation and evaluation of HIV treatment programs in the era of “Test and Treat” [67–69]. In decentralised settings in LMICs, technological and health system challenges occur in each step of the cascade. VL measurement is mostly done with laboratory assays, and fresh plasma is the preferred sample type. However, the cold chain system for blood sample transportation and 24-hour time window requirements preclude the use of this sample type in rural and remote areas. Dried blood spot (DBS) is an alternative sampling method, but may reduce the accuracy of VL measurement due to the detection of proviral DNA and intracellular RNA in whole blood samples, leading to over-quantification of viral load result [70]. It is noted that in the newly updated WHO guideline [71], a VL cut-off of 50 copies/ml was added on the treatment monitoring algorithm to identify PLHIV with low-level viremia (50-1000 copies/ml). This addition is likely to make the use of DBS for routine VL monitoring even more challenging as the limit of detection of 50 copies/ml, using this sampling method, is unlikely to be achieved with current VL testing platforms [72].

New sampling technologies [73, 74] have emerged, enabling accurate VL measurement by removing blood cells from the plasma component without the need for centrifugation. These are promising alternatives to the DBS as they could allow the detection of low-level viremia. However, further research is needed to determine the limit of detection, feasibility, and cost-effectiveness of these new devices/methods in real-world settings. New POC VL technologies have also become available and have the potential to decentralise VL testing. The use of these technologies, especially when VL results are more time-sensitive (such as VL results for breast feeding women and their children, people with an initial elevated VL) would be important to enable timely clinical decision and improve treatment outcomes. As HIV treatment programs mature, the number of PLHIV on ART will continue to increase, as well as the need for VL monitoring, second-line [75] and third-line ART [76], even in settings with lack of access to drug resistance testing. These challenges need to be addressed simultaneously and systematically to ensure the long-term effectiveness and efficacy of HIV treatment in LMICs.

## Limitation

This review had several limitations. Only peer-reviewed articles published in English were considered, meaning grey literature (unpublished and/or non-peer-reviewed and/or non-English national/program reports) with data on the outcomes of interest (e.g., annual PEPFAR monitoring and evaluation reports) was overlooked. The lack of data from geographic regions outside of SSA also raises a concern about bias and the generalizability of the findings. Another limitation is that some of the recently published studies report data collected 10–15 years ago which may not necessarily represent the recent uptake of VL monitoring. However, given the nature of the data reported from included studies (e.g., a wide range of VL coverage across study settings); these limitations are unlikely to affect the results and their interpretation substantially. Few studies reported a complete viral load cascade (number/proportion of patients with routine VL tests, unsuppressed VL, enhanced adherence counselling, follow-up VL and confirmed treatment failure). Researchers are encouraged to report on the viral load cascade of patients failing first-line ART and patients on second-line ART to enable understanding of the magnitude of the problem and identify intervention strategies and resources required to meet the needs of these vulnerable patients.

## Conclusions

Since 2016, there has been marked growth in the peer-reviewed literature on VL monitoring and VL cascade in PLHIV in LMICs. Most studies were conducted in SSA with significant financial and technical support from international/bilateral donors and implementing partners. Available data suggest significant gaps in VL monitoring services across countries and geographic regions, highlighting the needs to strengthen health system capacity for effective and sustainable implementation of routine VL monitoring for PLHIV who receive ART at decentralised care settings in LMICs. Particular attention is needed to rectify the failure cascade to ensure that VL monitoring and follow-up clinical actions are taken for individuals on ART who fail to achieve viral suppression. To advance the global agenda towards ending the HIV epidemic by 2030, we must fill the data gaps outside SSA; support the development and implementation of targeted interventions to improve VL monitoring and the VL cascade among populations at high risk of unsuppressed VL but with poor access to HIV treatment and care.

## Data Availability

All data generated or analysed during this study are included in the manuscript.

## Abbreviations

ART: antiretroviral therapy
DBS: Dried blood spot
EAC: Enhanced adherence counselling
KPs: Key populations
LMICs: Low and middle-income countries
PLHIV: People living with HIV
POC: Point of care
PMTCT: Prevention of mother to child transmission
SSA: sub-Saharan Africa
VL: Viral load

## References

[1] UNAIDS, 90-90-90: an ambitious treatment target to help end the AIDS epidemic. 2014, Joint United Nations Progamme on HIV/AIDS: Geneva, Switzeland. Available at https://www.unaids.org/en/resources/documents/2017/90-90-90. Accessed 14 July 2020.

[2] Quinn TC (2000). Viral load and heterosexual transmission of human immunodeficiency virus type 1. Rakai Project Study Group. N Engl J Med.

[3] Grinsztejn B (2014). Effects of early versus delayed initiation of antiretroviral treatment on clinical outcomes of HIV-1 infection: results from the phase 3 HPTN 052 randomised controlled trial. Lancet Infect Dis.

[4] Nachega JB (2018). Achieving Viral Suppression in 90% of People Living With Human Immunodeficiency Virus on Antiretroviral Therapy in Low- and Middle-Income Countries: Progress, Challenges, and Opportunities. Clin Infect Dis.

[5] Stover J (2016). What Is Required to End the AIDS Epidemic as a Public Health Threat by 2030? The Cost and Impact of the Fast-Track Approach. PLoS One.

[6] Bekker LG (2018). Advancing global health and strengthening the HIV response in the era of the Sustainable Development Goals: the International AIDS Society-Lancet Commission. Lancet.

[7] Charles B, Lalthanmawia R (2013). Providing HIV treatment closer to patient’s homes compared to more centralised treatment. Clin Epidemiol Global Health.

[8] Kredo T, et al. Decentralising HIV treatment in lower- and middle-income countries. Cochrane Database Syst Rev. 2013;(6):Cd009987. 10.1002/14651858.CD009987.pub2 PMC10009870 23807693

[9] Suthar AB (2014). Improving antiretroviral therapy scale-up and effectiveness through service integration and decentralization. AIDS.

[10] UNAIDS, Global HIV & AIDS statistics – 2020 Fact Sheet 2020, Joint United Nations Programme on HIV and AIDS: Geneva, Swizerland. Available at https://www.unaids.org/en/resources/fact-sheet. Accessed 10 July 2020.

[11] UNAIDS, How AIDS changed everything—MDG6: 15 years, 15 lessons of hope from the AIDS response. 2015, Joint United Nations Programme on HIV and AIDS Geneva. Switzerland. Available at https://www.unaids.org/en/resources/documents/2015/MDG6_15years-15lessonsfromtheAIDSresponse. Accessed 20 Dec 2019.

[12] Mutevedzi PC (2010). Scale-up of a decentralized HIV treatment programme in rural KwaZulu-Natal, South Africa: does rapid expansion affect patient outcomes?. Bull World Health Organ.

[13] Boyer S (2012). Performance of HIV care decentralization from the patient’s perspective: health-related quality of life and perceived quality of services in Cameroon. Health Policy Plan.

[14] Abongomera G (2018). Patient-level benefits associated with decentralization of antiretroviral therapy services to primary health facilities in Malawi and Uganda. Int Health.

[15] Haakenstad A (2019). Potential for additional government spending on HIV/AIDS in 137 low-income and middle-income countries: an economic modelling study. Lancet HIV.

[16] Resch S, Ryckman T, Hecht R (2015). Funding AIDS programmes in the era of shared responsibility: an analysis of domestic spending in 12 low-income and middle-income countries. Lancet Glob Health.

[17] Roberts T (2016). Scale-up of Routine Viral Load Testing in Resource-Poor Settings: Current and Future Implementation Challenges. Clin Infect Dis.

[18] Pham MD (2017). Feasibility of antiretroviral treatment monitoring in the era of decentralized HIV care: a systematic review. AIDS Res Ther.

[19] Marsh K (2019). Global, regional and country-level 90-90-90 estimates for 2018: assessing progress towards the 2020 target. AIDS.

[20] Moher D (2009). Preferred reporting items for systematic reviews and meta-analyses: the PRISMA statement. PLoS Med.

[21] WHO, Consolidated guidelines on the use of antiretroviral drugs for treating and preventing HIV infection: Recommendation for a public health approach - Second edition. 2016, The World Health Organization: Geneva, Switzerland. Available at https://www.who.int/hiv/pub/arv/arv-2016/en/. Accessed 20 Sept 2021.

[22] Iwuji CC (2020). Clinical outcomes after first-line HIV treatment failure in South Africa: the next cascade of care. HIV Med.

[23] Kehoe K (2020). Long-term virologic responses to antiretroviral therapy among HIV-positive patients entering adherence clubs in Khayelitsha, Cape Town, South Africa: a longitudinal analysis. J Int AIDS Soc.

[24] Le Roux KW, et al. A Case Study of an Effective and Sustainable Antiretroviral Therapy Program in Rural South Africa. AIDS Patient Care & Stds. 2019;33(11):466–472. 10.1089/apc.2019.0055 PMC6839417 31682167

[25] Euvrard J, et al. How accurately do routinely reported HIV viral load suppression proportions reflect progress towards the 90-90-90 target in the population on antiretroviral treatment in Khayelitsha, South Africa? South Afr Med J Suid-Afrikaanse Tydskrif Vir Geneeskunde. 2019;109(3):174–7. 10.7196/SAMJ.2019.v109i3.13456 30834874

[26] Copelyn J, et al. Short-term outcomes of down-referral in provision of paediatric antiretroviral therapy at Red Cross War Memorial Children’s Hospital, Cape Town, South Africa: A retrospective cohort study. South Afr Med J Suid-Afrikaanse Tydskrif Vir Geneeskunde. 2018;108(5):432–8. 10.7196/SAMJ.2018.v108i5.12855 29843859

[27] Tsondai PR (2017). High rates of retention and viral suppression in the scale-up of antiretroviral therapy adherence clubs in Cape Town, South Africa. J Int AIDS Soc.

[28] Haghighat R (2021). The HIV care cascade for adolescents initiated on antiretroviral therapy in a health district of South Africa: a retrospective cohort study. BMC Infect Dis.

[29] Woldesenbet SA, et al. Coverage of maternal viral load monitoring during pregnancy in South Africa: Results from the 2019 national Antenatal HIV Sentinel Survey. HIV Med. 2021;22(9):805–15. 10.1111/hiv.13126. Epub 2021 Jul 1. 10.1111/hiv.13126 PMC9292699 34213065

[30] Mshweshwe-Pakela N (2020). Feasibility of implementing same-day antiretroviral therapy initiation during routine care in Ekurhuleni District, South Africa: Retention and viral load suppression. South Afr J HIV Med.

[31] Sunpath H (2018). Targeting the third ‘90’: introducing the viral load champion. Public Health Action.

[32] Moyo F (2018). Near-real-time tracking of gaps in prevention of mother-to-child transmission of HIV in three districts of KwaZulu-Natal Province, South Africa. S Afr Med J.

[33] Tapera T (2019). Effects of a Peer-Led Intervention on HIV Care Continuum Outcomes Among Contacts of Children, Adolescents, and Young Adults Living With HIV in Zimbabwe. Glob Health Sci Pract.

[34] Nyakura J (2019). Viral load testing among women on ‘option B+’ in Mazowe, Zimbabwe: How well are we doing?. PLoS One.

[35] Nyagadza B (2019). Scaling up HIV viral load monitoring in Manicaland, Zimbabwe: challenges and opportunities from the field. Public Health Action.

[36] Apollo T (2021). Provision of HIV viral load testing services in Zimbabwe: Secondary data analyses using data from health facilities using the electronic Patient Monitoring System. PLoS One.

[37] Ndagijimana Ntwali JD (2019). Viral load detection and management on first line ART in rural Rwanda. BMC Infect Dis.

[38] Cyamatare Rwabukwisi F (2016). Five-year Outcomes Among Children Receiving Antiretroviral Therapy in a Community-based Accompaniment Program in Rural Rwanda. Pediatr Infect Dis J.

[39] Amzel A (2018). Community-Based Interventions to Reach 95-95-95 for Children and Adolescents: An Exploratory Programmatic Review From Lesotho. J Acquired Immune Deficiency Syndromes.

[40] Etoori D (2018). Challenges and successes in the implementation of option B + to prevent mother-to-child transmission of HIV in southern Swaziland. BMC Public Health.

[41] Cisse AM (2019). High level of treatment failure and drug resistance to first-line antiretroviral therapies among HIV-infected children receiving decentralized care in Senegal. BMC Pediatr.

[42] Nicholas S (2019). Point-of-care viral load monitoring: outcomes from a decentralized HIV programme in Malawi. J Int AIDS Soc.

[43] Moudachirou R (2020). Retention and sustained viral suppression in HIV patients transferred to community refill centres in Kinshasa, DRC. Public Health Action.

[44] Kadima J (2018). Adoption of routine virologic testing and predictors of virologic failure among HIV-infected children on antiretroviral treatment in western Kenya. PLoS One.

[45] Swannet S (2017). Journey towards universal viral load monitoring in Maputo, Mozambique: many gaps, but encouraging signs. Int Health.

[46] Namale G (2019). Sustained virological response and drug resistance among female sex workers living with HIV on antiretroviral therapy in Kampala, Uganda: a cross-sectional study. Sex Transm Infect.

[47] Nakalega R (2020). Non-uptake of viral load testing among people receiving HIV treatment in Gomba district, rural Uganda. BMC Infect Dis.

[48] Opito R (2020). Treatment outcome of the implementation of HIV test and treat policy at The AIDs Support Organization (TASO) Tororo clinic, Eastern Uganda: A retrospective cohort study. PLoS One.

[49] Brazier E, et al. Effects of national adoption of Treat-All guidelines on pre-ART CD4 testing and viral load monitoring after ART initiation: A regression discontinuity analysis. Clin Infect Dis. 2021;73(6):e1273–e1281. 10.1093/cid/ciab222. 10.1093/cid/ciab222 PMC8442775 33693517

[50] Herce ME (2020). Universal test-and-treat in Zambian and South African correctional facilities: a multisite prospective cohort study. Lancet HIV.

[51] Kyaw NT (2017). High rate of virological failure and low rate of switching to second-line treatment among adolescents and adults living with HIV on first-line ART in Myanmar, 2005–2015. PLoS One.

[52] Thinn KK (2019). Uptake of routine viral load testing among people living with HIV and its implementation challenges in Yangon region of Myanmar: a mixed-methods study. BMJ Open.

[53] Ya SST, et al. Performance and Outcomes of Routine Viral Load Testing in People Living with HIV Newly Initiating ART in the Integrated HIV Care Program in Myanmar between January 2016 and December 2017. Trop Med Infect Dis. 2020;5(3):140. 10.3390/tropicalmed5030140. 10.3390/tropicalmed5030140 PMC7557853 32878307

[54] Januraga PP (2018). The cascade of HIV care among key populations in Indonesia: a prospective cohort study. Lancet HIV.

[55] Nguyen TA (2020). Feasibility of dried blood spots for HIV viral load monitoring in decentralized area in North Vietnam in a test-and-treat era, the MOVIDA project. PLoS One.

[56] Ehrenkranz PD (2019). The missed potential of CD4 and viral load testing to improve clinical outcomes for people living with HIV in lower-resource settings. PLoS Med.

[57] Carmona S, Peter T, Berrie L (2017). HIV viral load scale-up: multiple interventions to meet the HIV treatment cascade. Curr Opin HIV AIDS.

[58] Han WM (2021). Global estimates of viral suppression in children and adolescents and adults on antiretroviral therapy adjusted for missing viral load measurements: a multiregional, retrospective cohort study in 31 countries. Lancet HIV.

[59] Sandbulte M (2020). Maternal viral load monitoring: Coverage and clinical action at 4 Kenyan hospitals. PLoS One.

[60] Yotebieng M (2019). HIV viral suppression among pregnant and breastfeeding women in routine care in the Kinshasa province: a baseline evaluation of participants in CQI-PMTCT study. J Int AIDS Soc.

[61] Cote AM (2004). Transactional sex is the driving force in the dynamics of HIV in Accra, Ghana. AIDS.

[62] Huerga H (2017). Higher risk sexual behaviour is associated with unawareness of HIV-positivity and lack of viral suppression - implications for Treatment as Prevention. Sci Rep.

[63] Vaz P (2020). Compromise of Second-Line Antiretroviral Therapy Due to High Rates of Human Immunodeficiency Virus Drug Resistance in Mozambican Treatment-Experienced Children With Virologic Failure. J Pediatric Infect Dis Soc.

[64] Kantor R (2018). HIV-1 second-line failure and drug resistance at high-level and low-level viremia in Western Kenya. AIDS.

[65] Pennings PS (2013). HIV Drug Resistance: Problems and Perspectives. Infect Dis Rep.

[66] Labhardt ND (2017). When patients fail UNAIDS’ last 90 - the “failure cascade” beyond 90-90-90 in rural Lesotho, Southern Africa: a prospective cohort study. J Int AIDS Soc.

[67] Etoori D (2018). Successes and challenges in optimizing the viral load cascade to improve antiretroviral therapy adherence and rationalize second-line switches in Swaziland. J Int AIDS Soc.

[68] Glass TR (2019). The viral load monitoring cascade in a resource-limited setting: A prospective multicentre cohort study after introduction of routine viral load monitoring in rural Lesotho. PLoS One.

[69] Ford N (2019). HIV viral resuppression following an elevated viral load: a systematic review and meta-analysis. J Int AIDS Soc.

[70] Smit PW (2014). Systematic review of the use of dried blood spots for monitoring HIV viral load and for early infant diagnosis. PLoS One.

[71] WHO, Consolidated guidelines on HIV prevention, testing, treatment, service delivery and monitoring: Recommendation for a public health approach. 2021, The World Health Organization: Geneva, Switzerland. Available at https://www.who.int/publications/i/item/9789240031593. Accessed 30 Mar 2022. 34370423

[72] WHO, HIV molecular diagnostics toolkit to improve access to viral load testing and infant diagnosis. 2019, The World Health Organization: Geneva, Switzerland. Available at https://www.who.int/publications/i/item/9789241516211. Accessed 30 Mar 2022.

[73] Pham MD, et al. Performance of a Novel Low-Cost, Instrument-Free Plasma Separation Device for HIV Viral Load Quantification and Determination of Treatment Failure in People Living with HIV in Malaysia: a Diagnostic Accuracy Study. J Clin Microbiol. 2019;57(4):e01683–18. 10.1128/JCM.01683-18. Print 2019 Apr. 10.1128/JCM.01683-18 PMC6440787 30700508

[74] Carmona S, et al. Separation of Plasma from Whole Blood by Use of the cobas Plasma Separation Card: a Compelling Alternative to Dried Blood Spots for Quantification of HIV-1 Viral Load. J Clin Microbiol. 2019;57(4):e01336–18. 10.1128/JCM.01336-18. Print 2019 Apr. 10.1128/JCM.01336-18 PMC6440768 30728197

[75] Estill J (2016). The need for second-line antiretroviral therapy in adults in sub-Saharan Africa up to 2030: a mathematical modelling study. Lancet HIV.

[76] Eholie SP (2019). Implementation of an intensive adherence intervention in patients with second-line antiretroviral therapy failure in four west African countries with little access to genotypic resistance testing: a prospective cohort study. Lancet HIV.

